# Venetoclax and alvocidib are both cytotoxic to acute myeloid leukemia cells resistant to cytarabine and clofarabine

**DOI:** 10.1186/s12885-020-07469-x

**Published:** 2020-10-12

**Authors:** Rie Nishi, Hiroko Shigemi, Eiju Negoro, Miyuki Okura, Naoko Hosono, Takahiro Yamauchi

**Affiliations:** 1 Department of Hematology and Oncology, Faculty of Medical Sciences, University of Fukui, 23-3 Shimoaizuki, Matsuoka, Eiheiji, Fukui, 910-1193 Japan; 2 Public Health Center of Tango, 855 Tanba, Mineyama, Kyotango, Kyoto, 627-8570 Japan

**Keywords:** Venetoclax, Bcl-2, Alvocidib, Mcl-1, Cytarabine (ara-C), Clofarabine (CAFdA)

## Abstract

**Background:**

Cytarabine (ara-C) is the major drug for the treatment of acute myeloid leukemia (AML), but cellular resistance to ara-C is a major obstacle to therapeutic success. The present study examined enhanced anti-apoptosis identified in 3 newly established nucleoside analogue-resistant leukemic cell line variants and approaches to overcoming this resistance.

**Methods:**

HL-60 human AML cells were used to develop the ara-C– or clofarabine (CAFdA)-resistant variants. The Bcl-2 inhibitor venetoclax and the Mcl-1 inhibitor alvocidib were tested to determine whether they could reverse these cells’ resistance.

**Results:**

A 10-fold ara-C-resistant HL-60 variant, a 4-fold CAFdA-resistant HL-60 variant, and a 30-fold CAFdA-resistant HL-60 variant were newly established. The variants demonstrated reduced deoxycytidine kinase and deoxyguanosine kinase expression, but intact expression of surface transporters (hENT1, hENT2, hCNT3). The variants exhibited lower expression of intracellular nucleoside analogue triphosphates compared with non-variant HL-60 cells. The variants also overexpressed Bcl-2 and Mcl-1. Venetoclax as a single agent was not cytotoxic to the resistant variants. Nevertheless, venetoclax with nucleoside analogs demonstrated synergistic cytotoxicity against the variants. Alvocidib as a single agent was cytotoxic to the cells. However, alvocidib induced G1 arrest and suppressed the cytotoxicity of the co-administered nucleoside analogs.

**Conclusions:**

Three new nucleoside analogue-resistant HL-60 cell variants exhibited reduced production of intracellular analogue triphosphates and enhanced Bcl-2 and Mcl-1 expressions. Venetoclax combined with nucleoside analogs showed synergistic anti-leukemic effects and overcame the drug resistance.

## Background

Cytarabine (1-β-D-arabinofuranosylcytosine; ara-C) is the major chemotherapeutic agent for acute myeloid leukemia (AML) [1, 2]. Remission induction chemotherapy consists of regular-dose 7-day administration of ara-C plus 3-day administration of daunorubicin or idarubicin. This regimen has been used for over 30 years as the gold standard [3], providing complete response (CR) rates of nearly 80%, but only 30–40% of patients achieve cure [4, 5]. Because the clinical efficacy of ara-C is not long-lasting, most patients deteriorate and do not respond to further chemotherapy regimens. Reversing the ineffectiveness of anticancer agents is indispensable [1].

Ara-C, a nucleoside analog, is taken up by leukemic cells via the membrane transporter human equilibrative nucleoside transporter 1 (hENT1) [6, 7]. Ara-C is intracellularly converted to the monophosphate form by deoxycytidine kinase (dCK) and then to the triphosphate form, ara-C 5′-triphosphate (ara-CTP), an intracellular active metabolite of ara-C, by pyrimidine kinases. Ara-CTP is inserted into nuclear DNA to inhibit DNA synthesis at the S-phase, triggering mitochondria-mediated apoptosis [8, 9].

Clofarabine (2-chloro-9-[2-deoxy-2-fluoro-β-D-arabinofuranosyl] adenine, CAFdA), a nucleoside analog similar to ara-C, is pharmacologically more advantageous than ara-C [10, 11]. CAFdA is transported by hENT1, hENT2, and human concentrative nucleoside transporter 3 (hCNT3). CAFdA is intracellularly attached to phosphates by dCK and deoxyguanosine kinase (dGK) to CAFdA 5′-triphosphate (CAFdATP). Similar to ara-C, CAFdATP is inserted into DNA, resulting in S-phase-specific cytotoxicity [12]. Currently, CAFdA is used widely to treat AML in relapsed/refractory settings [13].

Since 2017, eight new anti-AML agents have been incorporated into treatment regimens, after 30 years in which there had been no significant progress in AML chemotherapy [3]. One promising agent is venetoclax, which interferes with anti-apoptotic Bcl-2 [14, 15]. In our previous studies, cultured nucleoside analogue-resistant AML cell lines acquired an anti-apoptotic phenotype in vitro [16, 17]. Older Bcl-2 inhibitors such as YC137 and ABT737 induced apoptosis, thus overcoming the drug resistance of these cell lines. These results strongly suggested that Bcl-2 was an attractive target for AML chemotherapy, even though Bcl-2 was first found in a patient with follicular lymphoma [18]. In the present study, 3 new variants of the HL-60 human AML cell line that exhibited resistance to ara-C or CAFdA were established. Their drug-resistant nature was extensively investigated with regard to transporters, kinases, intracellular triphosphates, the cell cycle, and the mechanism of cell death. Bcl-2 and Mcl-1, both of which interfere with apoptosis, were found to be overexpressed in the new drug-resistant variants. The anti-apoptosis mechanism was targeted to overcome the drug resistance using venetoclax. The Mcl-1 inhibitor alvocidib was also evaluated in parallel. The findings provide further confirmation of the rationale for using anti-apoptosis-targeting agents in the context of relapsed/refractory AML.

## Methods

### Drugs

Venetoclax, alvocidib, gemcitabine, and cladribine were purchased from Selleck Chemicals (Houston, TX, USA). Ara-C and CAFdA were purchased from Sigma-Aldrich (St. Louis, MO, USA).

### Establishment of nucleoside analog-resistant cell lines

HL-60 human leukemia cells (JCRB Cell Bank, Osaka, Japan: JCRB0085) were kept in medium containing ara-C or CAFdA. The starting concentrations (1/100 of the 50% inhibitory concentration [IC_50_] values) were 5 nM for ara-C and 0.5 nM for CAFdA. Drug concentrations were escalated with time, and one ara-C-resistant variant clone (HL-60/ara-C10) and two CAFdA-resistant variant clones (HL-60/CAFdA4, HL-60/CAFdA30) were ultimately obtained by the limiting dilution method [19].

### Cell growth

Cell proliferation was evaluated using either direct counting or XTT assay (Roche Diagnostics, Indianapolis, IN, USA) [19]. IC_50_ values were determined by constructing growth curves. Combination index (CI) values were determined to evaluate drug-drug interactions (CalcySyn, Biosoft, Cambridge, UK) [20, 21].

### Calculation of ara-CTP and CAFdATP in the cells

The nucleotide pool was prepared from cells (1 × 10^6^/mL, 10 mL) after they had been incubated with either drug [22]. Intracellular ara-CTP and CAFdATP were calculated by HPLC [22, 23]. The nucleotide pool was obtained from treated or untreated cells [22], and the sample was analyzed on a TOSOH HPLC system (TOSOH Corp., Tokyo, Japan) equipped with a TSK gel DEAE-2SW column (TOSOH Corp.). The eluent was prepared with 0.06 M disodium hydrogen monophosphate (pH 6.9) with 20% acetonitrile by constant flow (0.7 mL/min), and at 269 nm (ara-CTP) or 254 nm (CAFdATP) at ambient temperature.

### Determination of kinases, nucleoside transporters, and apoptotic proteins

Kinases (dCK, dGK), transporters (hENT1, hENT2, hENT3, hCNT3), and apoptosis-related factors (Bcl-2, Mcl-1, Bad, Bim, Bax, Bak) were determined using Western blot analysis [16]. Mouse monoclonal anti-dCK (Abcam Cambridge, UK), rabbit polyclonal anti-dGK (Abcam), mouse monoclonal anti-hENT1 (Santa Cruz Biotechnology, Dallas, TX, USA), rabbit monoclonal anti-hENT2 (Abcam), rabbit polyclonal anti-hCNT3 (Abcam), rabbit polyclonal anti-Bcl-2 (Cell Signaling Technology, Beverly, MA, USA), rabbit polyclonal anti-Mcl-1 (Cell Signaling Technology), rabbit monoclonal anti-Bad (Cell Signaling Technology), anti-Bim (Cell Signaling Technology), anti-Bax (Cell Signaling Technology), anti-Bak (Cell Signaling Technology), and anti-actin (Sigma-Aldrich, St. Louis, MO, USA) antibodies were used as the primary antibodies. Anti-mouse IgG and horseradish peroxidase-conjugated anti-rabbit IgG (Amersham Biosciences, Bucks, UK) were used as the secondary antibodies. Band density was detected by an Image Quant LAS4000 mini apparatus (GE Healthcare, Uppsala, Sweden).

### Cell cycle determination

Treated or untreated cells stained with 20 μg/mL propidium iodide for 15 min were subjected to flow cytometric analysis using a FACS Canto II system (BD Bioscience, Franklin Lakes, NJ, USA).

### Apoptosis analyzed by flow cytometry

Apoptosis was determined based on annexin V positivity [17].

### DNA microarray

Total RNA was extracted by NucleoSpin RNA (MACHEREY- NAGEL GmbH & Co. KG, Düren, Germany) using an RNA purification protocol (≥ 100 ng). The cyanine3-labeled cRNA was synthesized by in vitro transcription using total RNA according to the recommended protocol (Agilent Technologies, Santa Clara, CA, USA). Fragmentation and hybridization (65 °C, 10 rpm,17 h) to a microarray were performed using cyanine3-labeled cRNA. After washing, a microarray image was acquired with a Surescan microarray scanner. Output RAW data and the QC report were digitized from array image data using Agilent Feature Extraction. A comparative analysis was performed using normalized data. All of these steps were done by Takara bio (Kusatu, Shiga, Japan).

## Results

### Establishment of nucleoside analog-resistant leukemic cell lines

One ara-C-resistant HL-60 variant (HL-60/ara-C10) and 2 CAFdA-resistant HL-60 variants (HL-60/CAFdA4, HL-60/CAFdA30) were established by the limiting dilution method after 6-month incubation with ara-C or 3- or 6-month incubation with CAFdA. HL-60/ara-C10 cells were more resistant to ara-C by 10-fold than HL-60 cells, whereas HL-60/CAFdA4 cells and HL-60/CAFdA30 cells were more resistant to CAFdA than HL-60 cells by 4-fold and 30-fold, respectively (Table 1). Analysis of the cross-resistance of these variant cell lines (Table 1) indicated that ara-C-resistant cells and CAFdA-resistant cells were cross-resistant to the other drug. These variants were also resistant to similar nucleoside analogs, including gemcitabine and cladribine (Table 1). The cross-resistant nature of these cell lines could be attributed to the intracellular activation pathway associated with these nucleoside analogs. Thus, 3 new resistant variants were successfully developed.

**Table 1:** Drug sensitivities

Drug	HL-60 nM	HL-60/ara-C10 nM (RR)	HL-60/CAFdA4 nM (RR)	HL-60/CAFdA30 nM (RR)
Ara-C	670	6900 (10)	> 1000	> 1000
CAFdA	60	359 (6)	260 (4)	1780 (30)
Gemcitabine	3	302 (101)	339 (113)	2089 (696)
Cladribine	68	6154 (91)	44,700 (657)	62,100 (913)
50 nM venetoclax + ara-C		2320		
50 nM venetoclax + CAFdA			120	690

The IC_50_ values were calculated by using the XTT assay. RR, relative resistance calculated as the ratio of the IC_50_ of HL-60/ara-C10, HL-60/CAFdA4, or HL-60/CAFdA30 cells relative to that of HL-60. 50 nM venetoclax + ara-C; Cells were incubated for 72 h with 50 nM venetoclax and with ara-C at different concentrations, 50 nM venetoclax + CAFdA; Cells were incubated for 72 h with 50 nM venetoclax and with CAFdA at different concentrations. *Ara-C* Cytarabine, *CAFdA* Clofarabine

### Intracellular ara-CTP and CAFdATP production

**Fig. 1:**
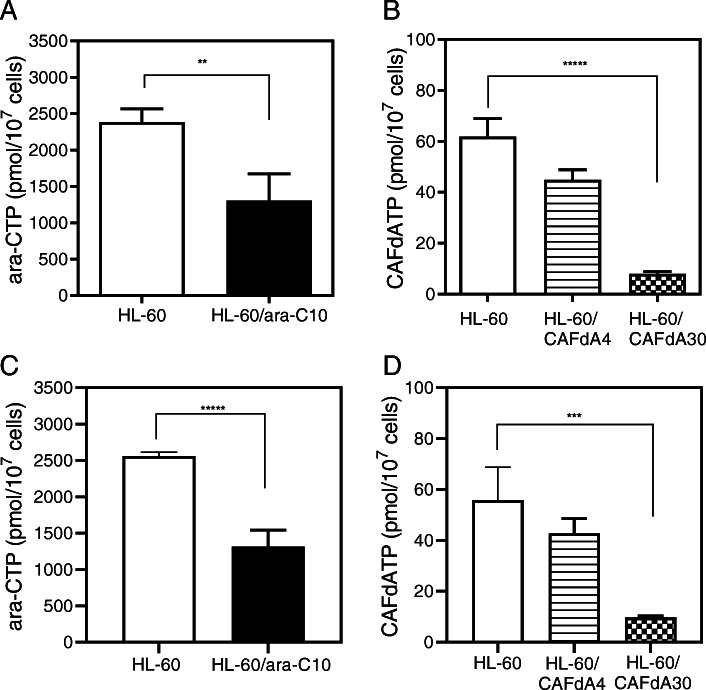
Intracellular ara-CTP (**a**, **c**) and CAFdATP (**b**, **d**) concentrations. Cells (1 × 10^6^ cells/mL, 10 mL) were incubated for 6 h with 10 μM ara-C or CAFdA and without (**a**, **b**) or with (**c**, **d**) venetoclax. Each value represents the mean ± SD of at least 3 independent experiments. a** *P* = 0.002.(**b**) ***** *P* = 0.0001. **c** *****P = 0.0003 . d*** *P* = 0.0035

The intracellular triphosphate form of a given nucleoside analog is crucial to its cell-killing activity [22]. When HL-60 cells were exposed to ara-C or CAFdA, the intracellular ara-CTP and CAFdATP concentrations were 2384 ± 183 pmol/1 × 10^7^ cells and 61.9 ± 7.1 pmol/1 × 10^7^ cells, respectively (Fig. 1a, b). However, the ara-CTP concentration was 1306 ± 368 pmol/1 × 10^7^ cells in HL-60/ara-C10 cells (HL-60 vs. HL-60/ara-C10, *P* = 0.002, *t*-test), whereas the CAFdATP concentration was 44.9 ± 4.0 pmol/1 × 10^7^ cells in HL-60/CAFdA4 cells (HL-60 vs. HL-60/CAFdA4, *P* = 0.01, *t*-test) and 8.0 ± 0.9 pmol/1 × 10^7^ cells in HL-60/CAFdA30 cells (HL-60 vs. HL-60/CAFdA30, *P* = 0.0001, *t*-test). Thus, levels of the intracellular analog triphosphates were significantly reduced in these resistant cell lines, and the reduction in the CAFdATP concentration was greater in the more-resistant HL-60/CAFdA30 cells.

### Transporter and kinase expression levels

**Fig. 2:**
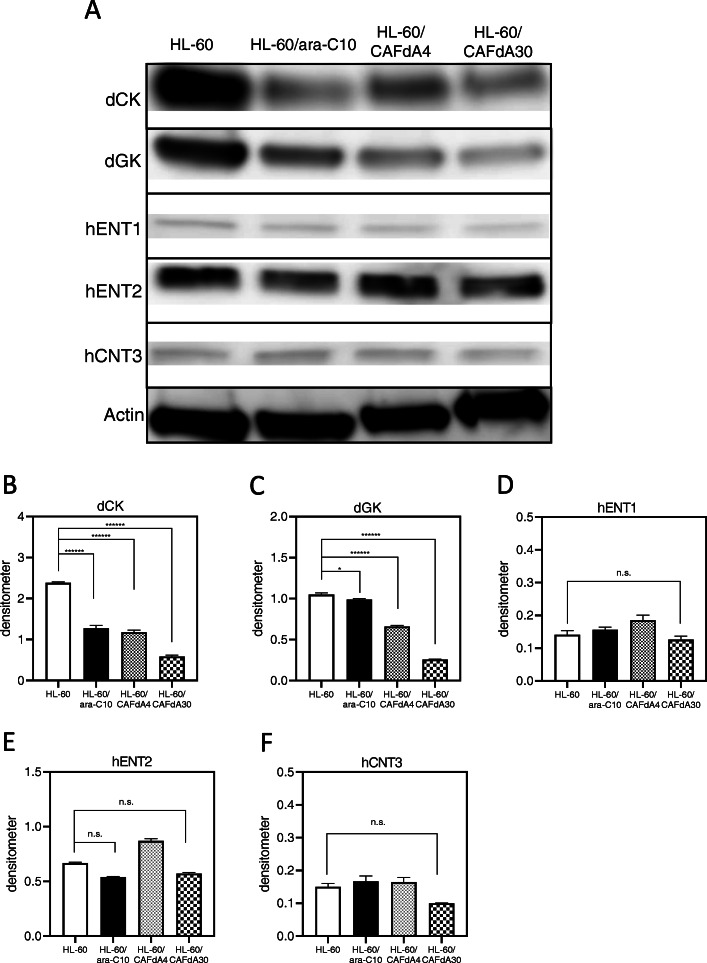
**a** Expression levels of kinases (dCK, dGK), transporters (hENT1, hENT2, hCNT3) were determined by Western blotting. These blots were cropped from the full length original blot (Supplementary Fig. 1A-F). The samples are derived from the same experiment, and the blots were processed in parallel. Band intensity values are shown below the corresponding band. Each value represents the mean ± SD of at least 3 independent experiments. Measurement with densitometer value, (**b**) dCK ******P < 0.0001 (HL-60 vs HL-60/ara-C10, HL-60/CAFdA4, HL-60/CAFdA30). **c** dGK *P = 0.012 (HL-60 vs HL-60/ara-C10), ******P < 0.0001 (HL-60 vs HL-60/CAFdA4, HL-60/CAFdA30). **d**,**e**,**f** hENT1, hENT2, hCNT3 *P* > 0.05. n.s., not significant

For successful triphosphate formation, ara-C must be taken up into cells via hENT1 and connected to phosphates by dCK, whereas CAFdA uses hENT1 and hCNT3 as transporters and is phosphorylated by both dCK and dGK [9, 11]. HL-60/ara-C10, HL-60/CAFdA4, and HL-60/CAFdA30 cells exhibited lower expression of dCK and dGK than HL-60 cells (Fig. 2a-c). The densitometric analyses demonstrated significant decreases in dCK and dGK in the 3 drug-resistant cell lines (Fig. 2b,c). In addition, we have already produced several nucleoside analog-resistant cell lines, in which protein levels, transcript levels, and kinase activities of dCK and dGK were reduced, indicating the contribution of reduced dCK and dGK to drug resistance [7, 9, 16, 17]. The degree of the reduction was greater in HL-60/CAFdA30 cells than in HL-60/CAFdA4 cells. There were no changes in hENT1, hENT2, and hCNT3 expressions in the 3 resistant variants (Fig. 2a,d-f). Moreover, microarray analyses demonstrated no changes in hENT1 and hCNT3 (Table 6). Thus, these results suggest that impaired intracellular phosphorylation of the drugs, rather than impaired drug transport, contributed to the observed reduction in analog triphosphate production.

### Expression of apoptosis-related proteins

**Fig. 3:**
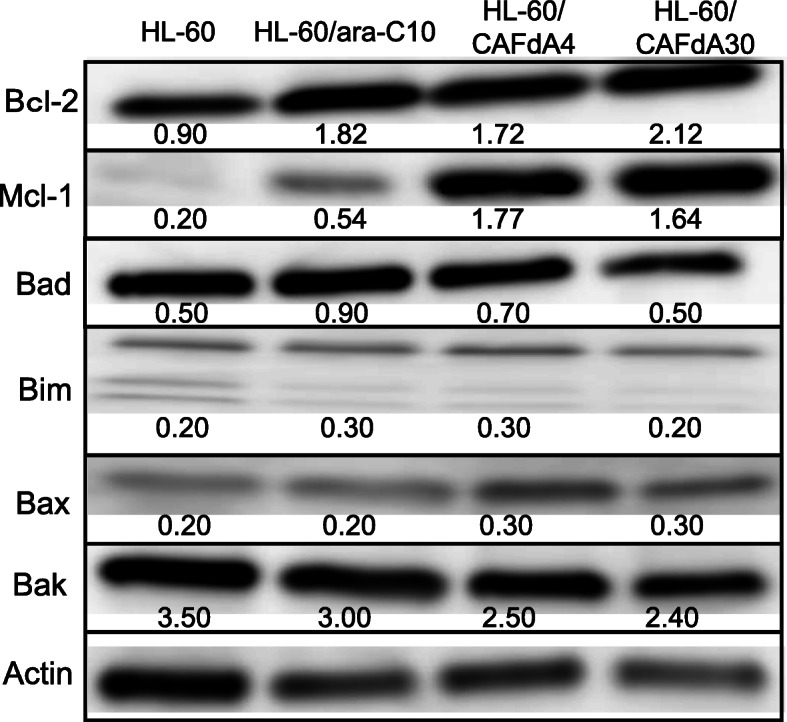
Expression levels of antiapoptotic (Bcl-2, Mcl-1) and proapoptotic (Bad, Bim, Bax, Bak) proteins were determined by Western blotting. These blots are cropped from the full length original blot (Supplementary Fig. 2A-G). The samples are derived from the same experiment, and the blots were processed in parallel. Band intensity values are shown below the corresponding band. Each value represents the mean ± SD of at least 3 independent experiments. Bcl-2, Mcl-1 *P* < 0.0001 (HL-60 vs HL-60/ara-C10, HL-60/CAFdA4, HL-60/CAFdA30)

The cytotoxicity of many anticancer agents involves the activation of apoptotic pathways [24]. The Bcl-2 family is directly associated with mitochondria-driven apoptosis, among which Bcl-2, Bcl-xL, and Mcl-1 are anti-apoptotic. Bcl-2 was originally discovered in follicular lymphoma with t (14;18), but its overexpression prevails in many types of cancers [18, 25]. Mcl-1, which is indispensable for the survival of multiple cell lineages, is frequently amplified in cancer cells, especially in the context of chemotherapy resistance [26]. By Western blotting, protein levels of anti-apoptotic Bcl-2 and Mcl-1 were increased in all three resistant cell lines (HL-60/ara-C10, HL-60/CAFdA4, HL-60/CAFdA30) compared with HL-60 cells (Fig. 3). Notably, higher Bcl-2 expression was seen in the cells more resistant to CAFdA (Fig. 3). Thus, these data suggest that anti-apoptosis was critical in the ara-C and CAFdA resistance mechanisms of these variants.

### Cytotoxicity of the Bcl-2 inhibitor venetoclax

**Fig. 4:**
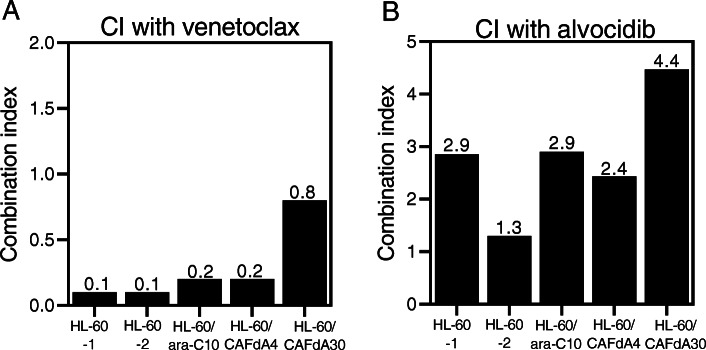
The CI in HL-60 and the 3 drug-resistant cell lines. The CI values were calculated after the cells had been exposed to nucleoside analogs (ara-C/CAFdA) or inhibitors (venetoclax/alvocidib) or both simultaneously. The CI values of less than, equal to, and more than 1 indicate synergy, additivity, and antagonism, respectively. **a** Combination of ara-C/CAFdA and venetoclax. HL-60-1; ara-C and venetoclax, HL-60-2; CAFdA and venetoclax, HL-60/ara-C10; ara-C and venetoclax, HL-60/CAFdA4; CAFdA and venetoclax, HL-60/CAFdA30; CAFdA and venetoclax. **b** Combination of ara-C/CAFdA and alvocidib. HL-60-1; ara-C and alvocidib, HL-60-2; CAFdA and alvocidib, HL-60/ara-C10; ara-C and alvocidib, HL-60/CAFdA4; CAFdA and alvocidib, HL-60/CAFdA30; CAFdA and alvocidib

**Fig. 5:**
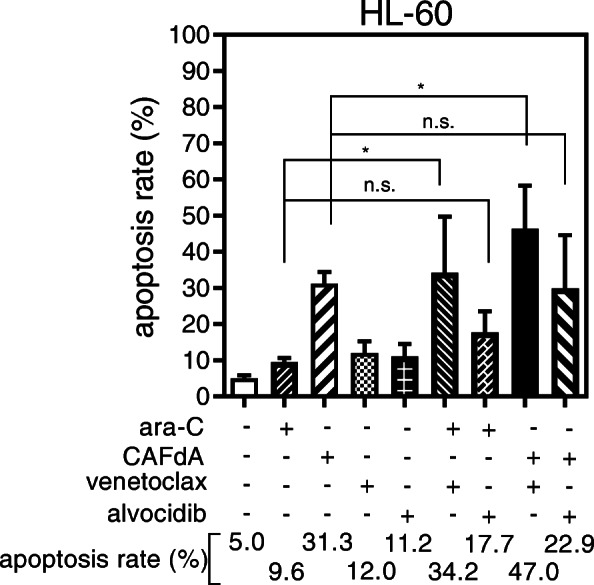
Apoptosis augmented by the inhibitors (venetoclax/alvocidib). HL-60 cells were treated for 24 h with 2 μM Ara-C or a minimally toxic concentration of venetoclax (5 nM)/alvocidib (50 nM) or both in combination. Alternatively, HL-60 cells were treated for 24 h with 1 μM CAFdA or a minimally toxic concentration of venetoclax (5 nM)/alvocidib (50 nM) or both simultaneously. Annexin V positivity was then analyzed by flow cytometry. Each value represents the mean ± SD of at least 3 independent experiments. *P = 0.022 (ara-C alone vs ara-C + venetoclax), *P = 0.017 (CAFdA alone vs CAFdA+venetoclax). n.s., not significant

**Fig. 6:**
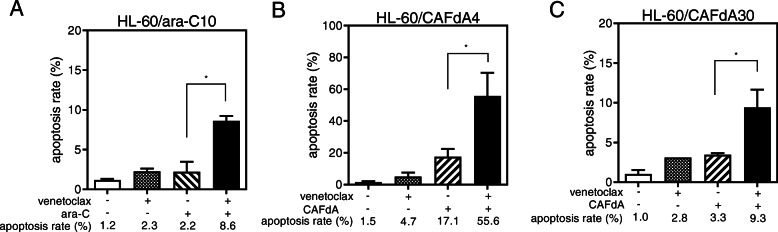
**a** The induction of apoptotic cell death by the addition of venetoclax. HL-60/ara-C10 cells were treated for 24 h with 2 μM ara-C or a minimally toxic concentration of venetoclax (50 nM) or both in combination. *P = 0.027. **b**, **c** HL-60/CAFdA4 cells (**b**) and HL-60/CAFdA30 cells (**c**) were treated for 24 h with 1 μM CAFdA or a minimally toxic concentration of venetoclax (50 nM) or both simultaneously. Annexin V positivity was then analyzed by flow cytometry. Each value represents the mean ± SD of at least 3 independent experiments. b*P = 0.017. c*P = 0.022

Bcl-2 is a potential chemotherapeutic target for certain cancers, typically those involving Bcl-2 overexpression. Venetoclax inhibits the interaction between the pro-apoptotic Bim BH3 peptide and Bcl-2, and it abrogates Bcl-2’s anti-apoptotic activity [25]. In the present study, venetoclax inhibited the growth of HL-60 cells, as well as the 3 drug-resistant variant lines (Table 2); however, the venetoclax IC_50_ values were higher for the drug-resistant cell lines than for HL-60 cells. This suggests that venetoclax alone is not sufficiently cytotoxic to overcome the chemoresistance of cells exhibiting enhanced Bcl-2 expression (Fig. 3, Table 2). The resistant cells (HL-60/ara-C10, HL-60/CAFdA4, HL-60/CAFdA30) were therefore treated with ara-C or CAFdA in combination with venetoclax, and the effect on growth was examined. The CI values were < 1 for all of the cell lines (0.1 for HL-60-1, 0.1 for HL-60-2, 0.2 for HL-60/ara-C10, 0.2 for HL-60/CAFdA4, 0.8 for HL-60/CAFdA30), suggesting synergism between the nucleoside analogs and venetoclax (Fig. 4a). The IC_50_ values decreased in the drug-resistant variants (HL-60/ara-C10, HL-60/CAFdA4, HL-60/CAFdA30) when the cells were incubated with different concentrations of ara-C or CAFdA in the presence of a minimal concentration of venetoclax (Table 1), suggesting a partial reversal of drug resistance. To further confirm the effect if the combination, a different cell line, MV4–11, was treated in a similar way (Table 3). The combination of ara-C and venetoclax showed greater inhibition of cell growth, compared with each agent alone. Apoptotic death of HL-60 cells treated with ara-C or CAFdA in the presence or absence of venetoclax was also quantified (Fig. 5). The combination of ara-C or CAFdA with venetoclax induced additive to more-than-additive apoptosis compared with each drug alone (Fig. 5). Similarly, the combination of ara-C or CAFdA with venetoclax induced additive to more-than-additive apoptosis in HL-60/ara-C10, HL-60/CAFdA4, and HL-60/CAFdA30 cells compared with each drug alone (Fig. 6). Importantly, ara-CTP and CAFdATP productions in HL-60 cells were unchanged (2560 ± 54 pmol/1 × 10^7^ cells and 55.8 ± 13.0 pmol/1 × 10^7^ cells, respectively) by co-incubation with venetoclax (Fig. 1c, d). By treatment with nucleoside analogs and venetoclax, the ara-CTP concentration was 1313 ± 228 pmol/1 × 10^7^ cells in HL-60/ara-C10 cells, whereas the CAFdATP concentrations were 42.8 ± 5.7 pmol/1 × 10^7^ cells in HL-60/CAFdA4 cells and 9.7 ± 0.7 pmol/1 × 10^7^ cells in HL-60/CAFdA30 cells (Fig. 1c, d). Thus, venetoclax enhanced the cytotoxicity of the nucleoside analogs and partially reversed the resistance of leukemic cells overexpressing Bcl-2, as venetoclax did not alter intracellular triphosphate production.

**Table 2:** The growth inhibition effects of venetoclax and alvocidib

Drug	HL-60 nM	HL-60/ara-C10 nM (RR)	HL-60/CAFdA4 nM (RR)	HL-60/CAFdA30 nM (RR)
Venetoclax	30	1200 (40)	2300 (77)	2400 (80)
Alvocidib	87	194 (2.2)	196 (2.3)	163 (1.9)

The IC_50_ were calculated by using the XTT assay. RR, relative resistance calculated as the ratio of the IC_50_ of HL-60/ara-C10, HL-60/CAFdA4, or HL-60/CAFdA30 cells relative to that of HL-60

**Table 3:** The growth inhibition effects of venetoclax and ara-C in MV4–11 cell line

Drug (nM)	MV4–11
Venetoclax	692
Ara-C	3055
Venetoclax+ara-C	1012

Cells were treated for 72 h with each agent at different concentrations,

followed by the determination of IC_50_ using the XTT assay

Venetoclax+ara-C; Cells were incubated for 72 h with 20 nM venetoclax

CI was 0.42

### Cytotoxicity of the Mcl-1 inhibitor alvocidib

Mcl-1 is also an anti-apoptotic factor known to be overexpressed in various cancers, including hematologic malignancies [27–29]. Alvocidib is an Mcl-1 inhibitor that inhibits the transcriptional activity of CDK9 to subsequently downregulate the transcription of target genes, including *MCL-1* [30]. In the present study, alvocidib inhibited the proliferation of HL-60 cells as well as the 3 drug-resistant variant cell lines (Table 2). The IC_50_ value was 2-fold higher than that in HL-60 cells, even in HL-60/CAFdA30 cells, suggesting that alvocidib as a single agent potently inhibits the growth of cells that overexpress Mcl-1 (Table 2). However, the CI values of HL-60, HL-60/ara-C10, HL-60/CAFdA4, and HL-60/CAFdA30 cells treated with ara-C or CAFdA and with alvocidib were all > 1, indicating antagonism (2.9 for HL-60-1, 1.3 for HL-60-2, 2.9 for HL-60/ara-C10, 2.4 for HL-60/CAFdA4, and 4.4 for HL-60/CAFdA30 cells) (Fig. 4b). Apoptotic death was quantified after HL-60 cells were incubated with ara-C or CAFdA and with or without alvocidib (Fig. 5). Neither ara-C nor CAFdA in combination with alvocidib showed enhancement of apoptosis induction. Treatment with the nucleoside analog (ara-C or CAFdA) in combination with alvocidib appeared to be less cytotoxic than combined treatment with venetoclax (*P* = 0.10 for ara-C + venetoclax vs. ara-C + alvocidib, *P* = 0.20 for CAFdA+venetoclax vs. CAFdA+alvocidib, *t*-tests). Thus, although Mcl-1 may have good chemotherapeutic potential, optimization of the use of alvocidib should be further considered.

### Cell cycle and drug scheduling

**Fig. 7:**
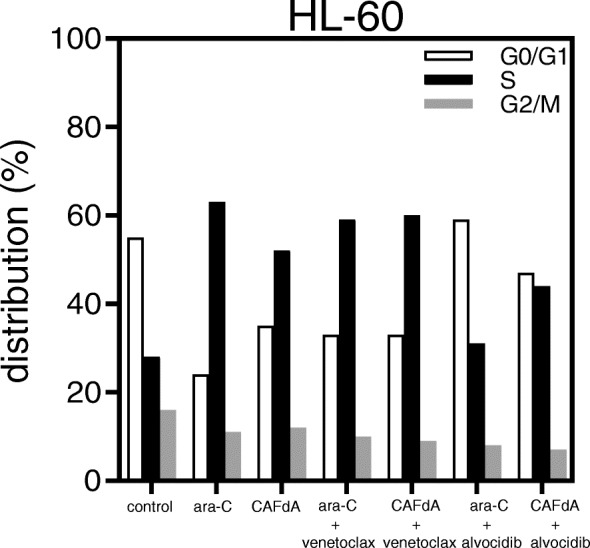
Cell cycle analysis. HL-60 cells were incubated for 24 h with ara-C or CAFdA, in the presence or absence of alvocidib, and subjected to flow cytometry

The cytotoxicity of nucleoside analogs derives from inhibition of DNA synthesis, which is therefore S-phase specific [9, 31]. Alvocidib reportedly inhibits CDK4 and CDK6, which regulate the cycling point, G1 - S [32–35]. HL-60 cells treated with ara-C or CAFdA accumulated in the S-phase (Fig. 7). Venetoclax did not reduce the fraction of the S phase where ara-C/CAFdA exerted its cytotoxicity. Treatment of cells with ara-C or CAFdA in combination with alvocidib increased in the G0/G1 phase and decreased in the S-phase compared with cells treated with ara-C or CAFdA alone (Fig. 7). These results suggest that the cytotoxicity of S-phase-specific nucleoside analogs is attenuated in combination with alvocidib. The IC_50_ of HL-60 cells pre-incubated with alvocidib for 24 h followed by a further 48-h incubation with the addition of ara-C was higher than that of cells incubated with ara-C alone (Table 4). In contrast, the IC_50_ of cells pre-incubated with ara-C for 24 h followed by a further 48-h incubation with the addition of alvocidib was lower than that of cells incubated with ara-C alone. Similar results were obtained with CAFdA and alvocidib (Table 4). These data thus suggest that the cytotoxicity of alvocidib is schedule dependent.

**Table 4:** IC_50_ values of alvocidib in HL-60 cells

Drug (nM)	IC _50_ values in HL-60
Alvocidib ➔ ara-C	989
Ara-C ➔ Alvocidib	380

Alvocidib ➔ ara-C; 50 nM alvocidib was added 24 h before ara-C,

Ara-C ➔ Alvocidib; ara-C was added 24 h before 50 nM alvocidib

### DNA microarray analyses

Microarray analyses were performed, and genes altered commonly in the 3 drug-resistant cell lines were identified. Among them, RRAS2 (Ras-related protein), KITLG (the ligand of the tyrosine-kinase receptor encoded by the KIT locus), MAP 3 K6 (mitogen-activated protein kinase kinase kinase 6), FGFR1 (mutation of this gene is associated with stem cell myeloproliferative disorder and stem cell leukemia lymphoma syndrome), MAPT (promotes microtubule assembly and stability), FLAT4 (fms-like tyrosine kinase 4 that promotes proliferation and survival), EFNA4 (associated with RAS pathway), and LAMC1 (laminin subunit gamma 1 mediating the attachment, migration, and organization of cells), were increased by greater than 2-fold in the 3 resistant cell lines (Table 5). Therefore, several survival pathways and oncogenes were also enhanced in the resistant cell lines, which would not be the targets of venetoclax (Table 5). Furthermore, microarray analyses demonstrated no changes in hENT1, hCNT3, and NF-kB1 (Table 6).

**Table 5:** Altered genes in the drug-resistant variants determined by DNA microarray analysis

Upregulated					Downregulated				
Gene	Ref. sequence	Fold change			Gene	Ref. sequence	Fold change		
		HL-60/ Ara-C10	HL-60/ CAFdA4	HL-60/ CAFdA30			HL-60/ Ara-C10	HL-60/ CAFdA4	HL-60/ CAFdA30
CDK7	NM_001799	3.12	2.83	2.83	CDA	NM_001785	0.46	0.47	0.49
CCND1	NM_053056	9.51	11.7	10.4	BCL2L11	NM_207002	0.35	0.35	0.31
CCND2	NM_001759	4.0	4.0	4.1	JAK2	NM_001322194	0.32	0.26	0.22
CCNB1	NM_031966	3.23	3.10	3.0	STAT5A	NM_003152	0.05	0.16	0.13
CCNB3	NM_033031	2.91	3.76	4.06	SOCS6	NM_004232	0.40	0.37	0.26
HDAC2	NM_001527	3.89	3.68	4.08	PTPN2	NM_002828	0.40	0.40	0.40
E2F5	NM_001951	4.32	3.89	4.32	ERN1	NM_001433	0.44	0.47	0.47
RRAS2	NM_012250	5.46	5.82	5.74					
MAP 3 K8	NM_005204	2.08	2.17	2.51					
KITLG	NM_000899	488	410	425					
MAP 3 K6	NM_004672	31.8	23.1	21.1					
FGFR1	NM_023110	580	498	530					
MAPT	NM_016835	125	97.0	105					
FLAT4	NM_002020	5.82	6.23	6.23					
EFNA4	NM_182690	6.36	6.54	7.78					
LAMC1	NM_002293	440	360	393					
PKN3	NM_013355	47.5	49.5	48.2					

There were 4804 genes that had variation compared to the parent strain in 60,901 total genes. Gene symbol were CDA: cytidine deaminase, BCL2L11: Bim

**Table 6:** Unmodified genes in the drug-resistant variants determined by DNA microarray analysis

Gene	Ref. sequence	Fold change		
		HL-60/Ara-C10	HL-60/CAFdA4	HL-60/CAFdA30
SLC29A1	NM_022127	1.31	1.27	1.29
SLC28A3	NM_001078	0.86	0.85	0.82
NFKB1	NM_003998	0.54	0.50	0.53

There were 4804 genes that had variation compared to the parent strain in 60,901 total genes

Gene symbol were SLC29A1:hENT1, SLC28A3: hCNT3

## Discussion

The combination of ara-C with daunorubicin or idarubicin has been a gold standard for the treatment of AML. This regimen provides CR in 70–80% of younger AML patients [4, 5]. Despite this high remission rate, most patients subsequently relapse with the development of drug resistance. To improve long-term outcomes in AML, new therapeutic modalities or strategies that overcome the development of resistance are needed. The Bcl-2 inhibitor venetoclax is one such promising agent. Venetoclax has exhibited potent anti-leukemic activity in both chronic lymphocytic leukemia and AML [36, 37]. However, the efficacy of incorporating venetoclax into anti-AML chemotherapies has not yet been fully confirmed in the context of drug resistance.

We have developed numerous drug-resistant cell line variants from cultured cancer cell lines [17, 19, 38–40]. Mechanisms of drug resistance thus far elucidated include alteration of cell surface transporters, impaired enzymes associated with intracellular drug metabolism, effects on DNA repair functions, cell cycle distribution, amplification and/or mutations in targeted genes, and apoptosis pathways. We observed enhanced anti-apoptosis in our previously established AML cell lines resistant to nucleoside analog, which therefore suggests anti-apoptosis as a chemotherapeutic target.

Our previous study demonstrated that the Bcl-2 inhibitor YC137 is cytotoxic to ara-C-resistant leukemic cells [16]. ABT737, which inhibits Bcl-2 and Bcl-xL, effectively induced death in CAFdA-resistant leukemic cells [17]. YC137, a naphthoquinone compound, binds to and inhibits the interaction of Bcl-2 with Bid, resulting in suppression of the anti-apoptotic activity of Bcl-2 and induction of apoptotic cell death [41]. However, YC137 has not been investigated clinically. ABT737 is one of the most powerful inhibitors known, capable of inhibiting the binding of BH3 peptide to Bcl-2 and Bcl-xL. ABT737 has not been evaluated in clinical trials, however, as its structure is not amenable to preparation of a form for oral administration. ABT263 (navitoclax), an orally administered active Bcl-2 and Bcl-xL inhibitor, is a derivative of ABT737 that has entered into clinical trials instead. Nevertheless, ABT263 was deemed unsuitable because it had a side effect of reducing the peripheral platelet level [42]. Thus, all agents that inhibited anti-apoptosis have been unsuccessful before venetoclax.

In the present study, one ara-C-resistant variant cell line and two CAFdA-resistant variant cell lines were newly established (Table 1). The major mechanism of resistance involved decreased production of intracellular triphosphate forms (ara-CTP, CAFdATP) (Fig. 1), primarily attributed to reduced expression of kinases (dCK, dGK) rather than transporters (Fig. 2a-c and Table 5). The amounts of CAFdATP of HL-60/CAFdA4 and of HL-60/CAFdA30 were 44.9 and 8.0 pmol/1 × 10^7^ cells, respectively. This is a nearly 5-fold difference between their values, which did not appear to be a very large difference. The degree of the difference in the values was similar to the difference in the degree of drug resistance between the HL-60/CAFdA4 and HL-60/CAFdA30 cell lines (4 vs 30). The production of CAFdATP depends on both dCK and dGK; dCK values were 1.18 for HL-60/CAFdA4 and 0.59 for HL-60/CAFdA30 (Fig. 2b), whereas, dGK values were 0.66 for HL-60/CAFdA4 and 0.26 for HL-60/CAFdA30 (Fig. 2c). Thus, more reduced kinases produced less CAFdATP in HL-60/CAFdA30 cells than in HL-60/CAFdA4 cells. The importance of the kinases was demonstrated previously in studies of cellular resistance to nucleoside analogs [7, 17, 39, 43, 44] and found to be associated with therapeutic outcomes [43, 45, 46]. Previously established nucleoside analog-resistant cell lines showed reduced cell surface hENT1 expression [16, 17]. The decrease in transporter expression prevented influx of the drugs into leukemic cells. The transporters of the cell lines examined here were not affected, but the reduced expression of kinases (dCK, dGK) led to insufficient metabolism of the drugs and subsequent production of only small amounts of ara-CTP/CAFdATP intracellularly (Fig.1a,b and Fig. 2). Although drug resistance mechanisms are usually multifactorial, the mechanistic contribution varies among cell variants with acquired resistance.

Another important mechanism of drug resistance involves enhanced anti-apoptosis. Augmentation of anti-apoptosis was again demonstrated in the present study in the newly developed nucleoside analog-resistant cell lines (HL-60/ara-C10, HL-60/CAFdA4, HL-60/CAFdA30) (Table 1, Fig. 3). Venetoclax was less cytotoxic to cells overexpressing Bcl-2 than to the parental HL-60 cells (Table 2). However, the combination of venetoclax and one of the nucleoside analog showed synergistic inhibition of cell growth (Fig. 4a) and induced additive to more-than-additive apoptosis (Figs. 5 and 6). This result could be attributed to a need for the activation of apoptosis by anticancer drugs before venetoclax suppresses the anti-apoptotic effect of Bcl-2 and kills cells. This in vitro result was in accordance with the efficacy of single administration of venetoclax in a previous clinical study [47], in which the overall response rate was only 19% in relapsed/refractory AML. Accordingly, venetoclax should be used with other anti-leukemic agents to improve its clinical efficacy. DiNardo et al. reported that venetoclax plus azacitidine or decitabine achieved a response rate of 67%, with a median overall survival of 17.5 months in newly diagnosed elderly AML patients [14]. The Cmax of venetoclax at the standard dose (200 mg) is 1.15 μg/mL (=1.3 μM). Moreover, the drug is administered to a patient every day. In the present study, venetoclax was used at nanoM levels, and only for the incubation duration of 24–72 h (Table 1). Therefore, the concentrations used in the present study are clinically achievable, and drug toxicity will not increase. One important finding of the present study was that venetoclax did not impair the production of intracellular active metabolites, nucleoside triphosphates (ara-CTP, CAFdATP) (Fig. 1c,d). Azacytidine and decitabine are both ara-C derivatives that are activated to the corresponding deoxyazacytidine triphosphate form via the same pathway.

It is very difficult to uncover the mechanism of the synergistic antileukemic activity between ara-C/CAFdA and venetoclax (Fig. 4a). As shown in Figs. 1 c, d, the production of intracellular triphosphate forms of cytarabine and clofarabine was not reduced by the addition of venetoclax. Further, venetoclax did not reduce the fraction of the S phase where ara-C and CAFdA exerted their cytotoxicity (Fig. 7). Ara-C and CAFdA effectively and apparently induced greater amounts of apoptosis via the inhibition of overexpressed Bcl-2 by co-incubation with venetoclax (Fig. 5). Nevertheless, microarray analyses were performed in an attempt to examine the mechanism of this synergy (Table 5). Commonly altered genes were identified in the 3 drug-resistant cell lines. Among them, several survival pathways and oncogenes were enhanced in the resistant cell lines, which were not the targets of venetoclax. This also means that to block Bcl-2 by venetoclax alone would not be cytotoxic enough for the resistant cell lines. Therefore, the synergism between venetoclax and nucleoside analogs would be attributable to: venetoclax did not have negative impacts on the mechanism of action of nucleoside analogs; the difficulty to induce apoptosis by ara-C/CAFdA due to overexpression of Bcl-2 was partially removed by venetoclax; and the inhibition of Bcl-2 by venetoclax as a single agent was not enough to induce cell death in the drug-resistant cells, which may require the cellular damage induced by ara-C/CAFdA.

Mcl-1 is a member of the Bcl-2 family, and its expression is reported to be increased in chemoresistant AML cells [30, 48]. Mcl-1 augmentation was identified as playing a critical role in the mechanism of resistance to Bcl-2 inhibitors [49], showing a poor prognosis and chemoresistance [29]. The resistant cell lines (HL-60/ara-C10, HL-60/CAFdA4, HL-60/CAFdA30) established in the present study also overexpressed Mcl-1 (Fig. 3). Although our findings did not show a relationship between Bcl-2 and Mcl-1, they suggested that both anti-apoptotic factors contribute to drug resistance. The Mcl-1 inhibitor alvocidib was investigated in parallel. Unlike venetoclax, alvocidib as a single agent was cytotoxic to the drug-resistant variants (HL-60/ara-C10, HL-60/CAFdA4, HL-60/CAFdA30) despite the overexpression of Mcl-1 (Table 2). However, alvocidib did not improve the cytotoxicity of ara-C or CAFdA (Fig. 4b), but, instead, it was rather antagonistic. Alvocidib inhibits not only CDK9 but also CDK4 and CDK6, which increase with cell cycle arrest at G1 [50, 51]. Figure 7 clearly demonstrates the G1 accumulation after treatment with alvocidib. Because the cytotoxicity of nucleoside analogs is S-phase-specific, G1 arrest induced by alvocidib would eventually inhibit the cytotoxicity of ara-C and CAFdA (Fig. 4b, Fig. 5, Table 4). Ara-C should thus be administered before alvocidib inhibits the cell cycle to improve the effect of this combination (Table 4).

Recently, various gene mutations have been reported to be responsible for treatment resistance of AML. Kirtonia et al. reviewed genetic alterations and molecular targeted therapies in the field of AML [52]. Molecular abnormalities of AML include *NPM1, CEBPα, RUNX1, DNMT3A, TET2, IDH1/2, FLT3, KIT, NRAS, WT1, TP53, PTPN11, U2AF1, SMC1A, SMC3, STAG2, RAD21, ASXL1/2,* and *EZH2,* which are mutated in more than 5% of AML cases. Several of these mutated genes are now targets for molecular targeted agents including enasidenib, ivosidenib, midostaurin, gilteritinib, and quizartinib. Moreover, upregulation of *PLK1, Bcl-2*, and Hedgehog signaling is detected in AML cells. Venetoclax and glasdegib have also been introduced to the clinic recently. Moreover, *FLT3* mutation is the most frequent mutation in AML patients. Garg et al. investigated *FLT3*-mutated AML cells from 80 patient samples and identified a number of novel driver genes. Importantly, it was suggested that there were two types of relapse, occurring from founder clones and from a subclone. In addition, purine-pyrimidine transversion mutations were more frequently seen at relapse after treatment using ara-C and daunorubicin [53]. Therefore, therapeutic strategies should be optimized and individualized based on genetic abnormalities, especially at the time of relapse. Therefore, selection of drugs targeting these causative factors of treatment resistance and the effects of combinations with other drugs must be examined.

Furthermore, Siveen et al. demonstrated that thymoquione abrogated NF-kB-regulated gene products in multiple myeloma cells [54]. In their study, thymoquione combined with bortezomib significantly inhibited NF-κB DNA-binding activity, which was due to the reduction in NF-kB phosphorylation. Moreover, Bcl-2, regulated by NF-kB, was also downregulated when treated with thymoquinone and bortezomib. In the present study, microarray analyses demonstrated no increases in NF-kB in the 3 drug-resistant cell lines (Table 6). Therefore, unlike myeloma cells, this combination treatment might not alter the activation of the NF-kB signaling cascade. However, it is necessary to consider the NF-kB signaling pathway that regulates Bcl-2 in the mechanism of action of this combination.

## Conclusion

The present study established one new ara-C-resistant and two CAFdA-resistant leukemic cell lines exhibiting impaired production of intracellular triphosphates and enhanced anti-apoptosis via Bcl-2 and Mcl-1. The Bcl-2 inhibitor venetoclax demonstrated synergism with nucleoside analogs and partially reversed the resistance in cells overexpressing Bcl-2. The Mcl-1 inhibitor alvocidib was cytotoxic to the cells, but the effect of its combination with nucleoside analogs was schedule-dependent. Anti-apoptosis is thus a clinical target of AML chemotherapy. Venetoclax combined with a nucleoside analog (azacytidine, ara-C) resulted in higher remission rates and longer survival than the nucleoside analog alone in AML [14, 55]. The combination of venetoclax and ara-C was evaluated in newly diagnosed AML patients who were not eligible for intensive chemotherapy in a phase 3 clinical study including our institution [55]. The results showed that the complete remission rate of ara-C plus venetoclax was 48%, which was much higher than that of ara-C alone (13%). Median overall survival was 7.2 months for ara-C plus venetoclax and 4.1 months for ara-C alone [55]. This combination is likely to be one of the standard regimens for relapsed AML, especially in elderly patients, in the very near future. The present in vitro basic experimental data will provide a rationale for its clinical efficacy. The findings of the present investigation could serve as the basis for optimizing chemotherapeutic strategies to obtain better clinical outcomes in the treatment of AML.

## Supplementary information

**Additional file 1.**

## Data Availability

Not applicable.

## Abbreviations

AML: Acute myeloid leukemia
CR: Complete remission
ara-C: Cytarabine
ara-CTP: Ara-C 5′-triphosphate
CAFdA: Clofarabine
CAFdATP: CAFdA 5′-triphosphate
hENT1: Human equilibrative nucleoside transporter 1
hENT2: Human equilibrative nucleoside transporter 2
hCNT3: Human concentrative nucleoside transporter 3
dCK: Deoxycytidine kinase
dGK: Deoxyguanosine kinase
CI: Combination index
IC_50_: 50% Inhibitory concentration
