# Supplementary material for: Venetoclax and alvocidib are both cytotoxic to acute myeloid leukemia cells resistant to cytarabine and clofarabine
Source: BMC Cancer. 2020 Oct 12;20:984. doi: 10.1186/s12885-020-07469-x (PMC7552348; doi:10.1186/s12885-020-07469-x)
Supplement: Supplementary file 1 — Additional file 1. [file 12885_2020_7469_MOESM1_ESM.pptx]

## Slide 1
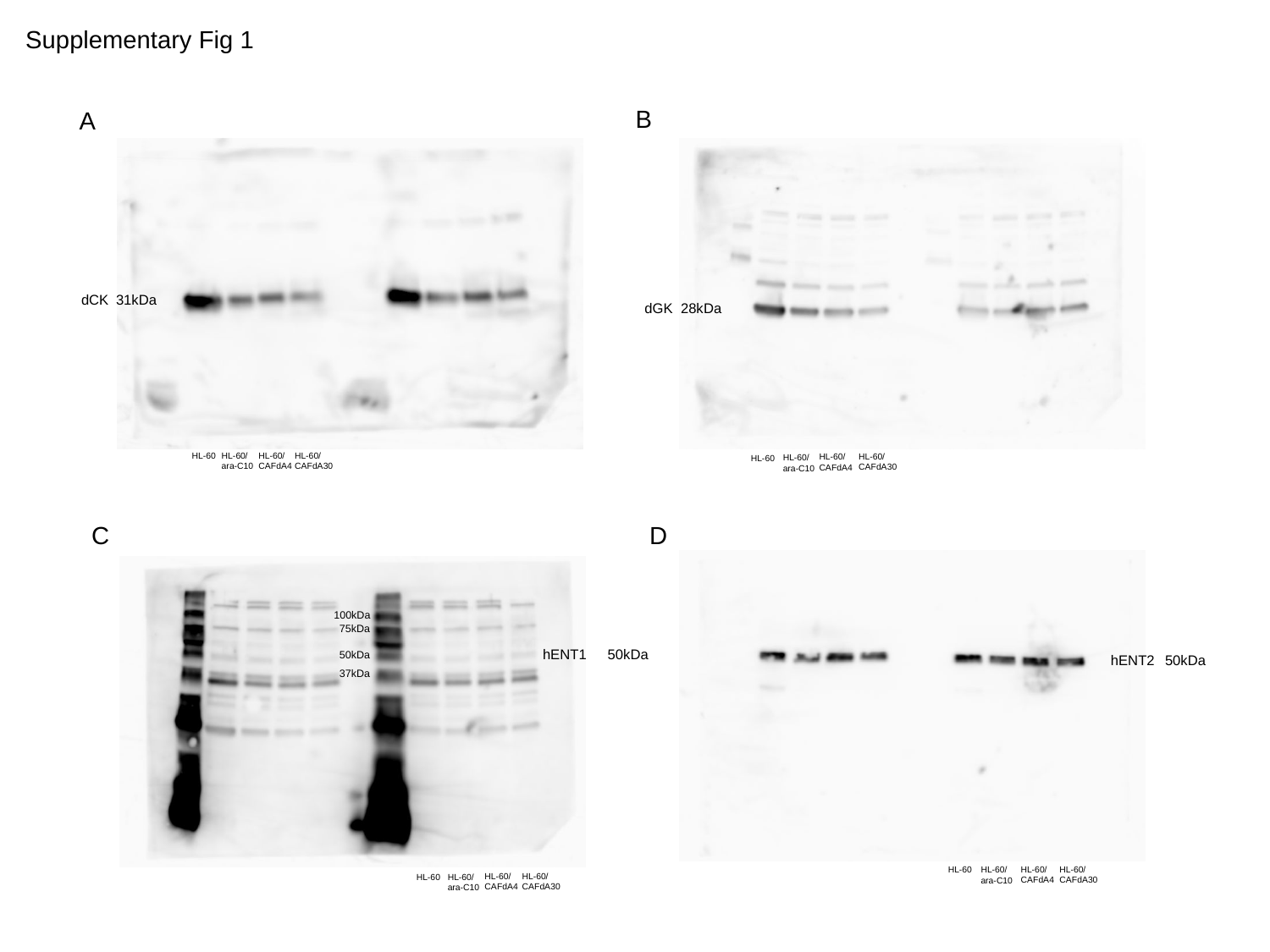

Supplementary Fig 1
B
A
dCK
31kDa
dGK
28kDa
HL-60/
CAFdA30
HL-60/
ara-C10
HL-60/
CAFdA4
HL-60
HL-60/
CAFdA30
HL-60/
CAFdA4
HL-60/
ara-C10
HL-60
D
C
100kDa
75kDa
hENT1　50kDa
50kDa
hENT2
50kDa
37kDa
HL-60/
CAFdA4
HL-60/
CAFdA30
HL-60
HL-60/
ara-C10
HL-60/
CAFdA30
HL-60/
CAFdA4
HL-60
HL-60/
ara-C10

## Slide 2
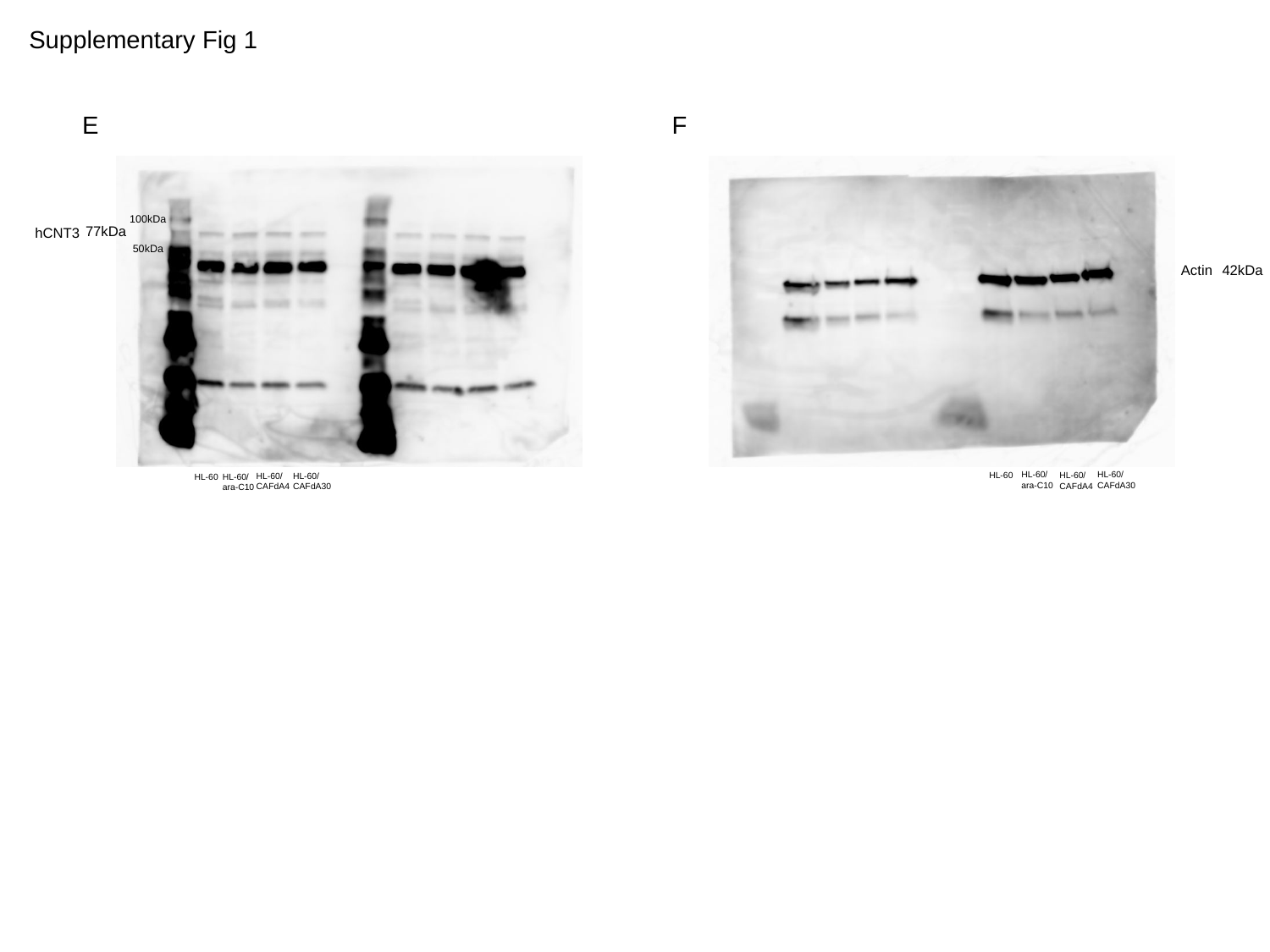

Supplementary Fig 1
E
F
100kDa
77kDa
hCNT3
50kDa
Actin
42kDa
HL-60/
CAFdA30
HL-60/
ara-C10
HL-60
HL-60/
CAFdA4
HL-60/
CAFdA30
HL-60/
CAFdA4
HL-60/
ara-C10
HL-60

## Slide 3
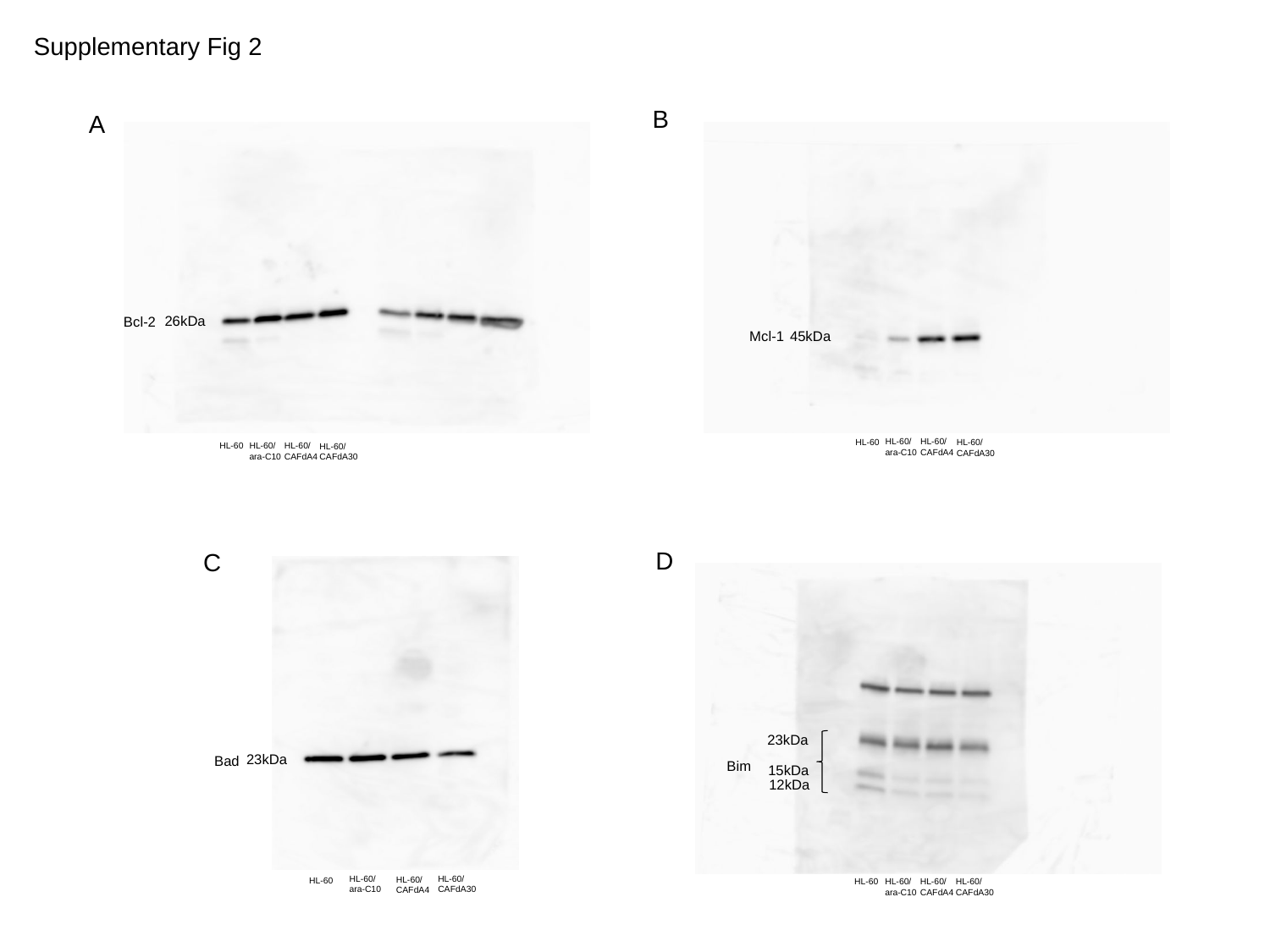

Supplementary Fig 2
B
A
26kDa
Bcl-2
Mcl-1
45kDa
HL-60/
ara-C10
HL-60/
CAFdA4
HL-60
HL-60/
CAFdA30
HL-60
HL-60/
CAFdA4
HL-60/
ara-C10
HL-60/
CAFdA30
D
C
23kDa
23kDa
Bad
Bim
15kDa
12kDa
HL-60/
ara-C10
HL-60/
CAFdA30
HL-60/
CAFdA4
HL-60
HL-60
HL-60/
ara-C10
HL-60/
CAFdA4
HL-60/
CAFdA30

## Slide 4
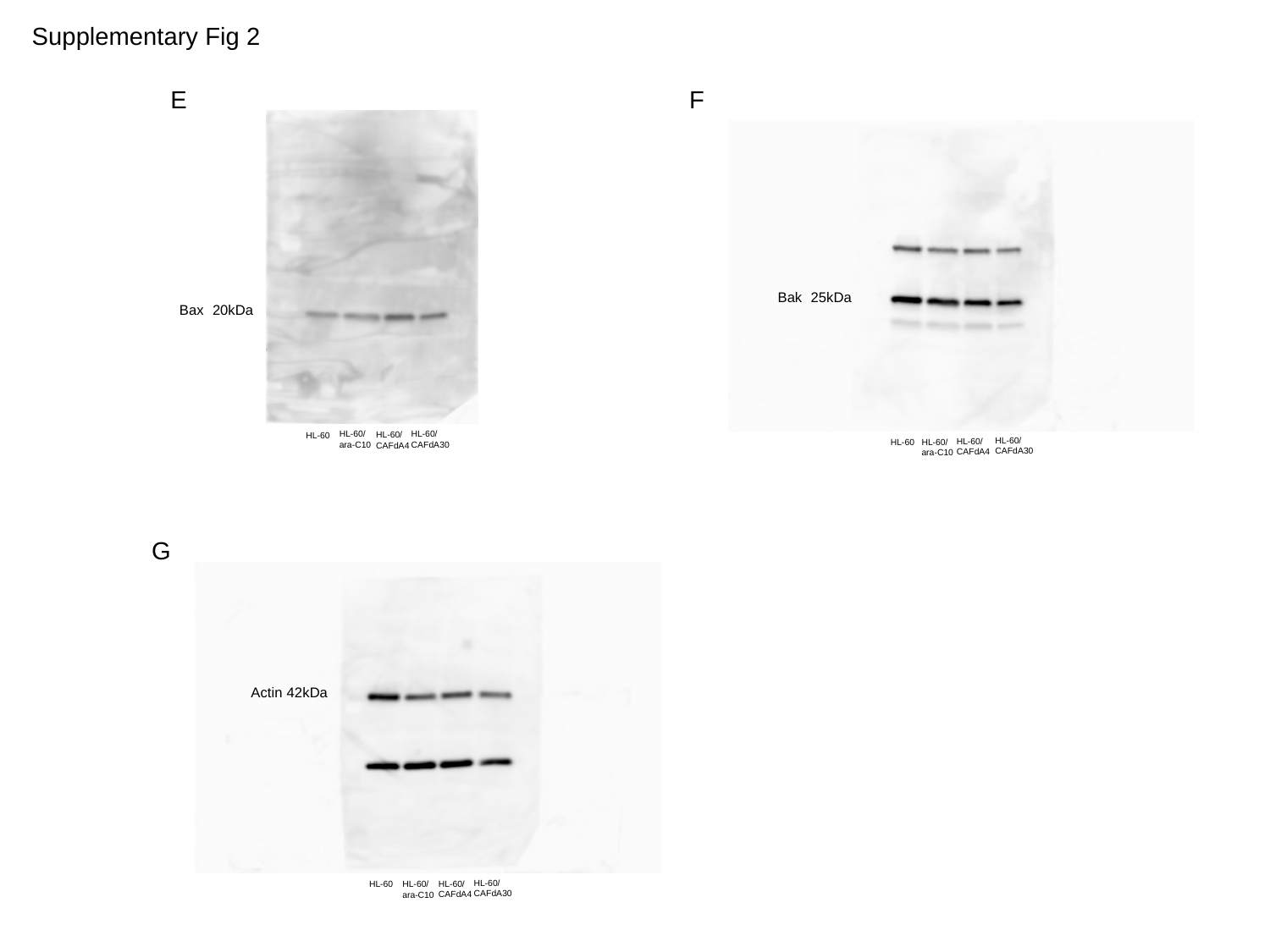

Supplementary Fig 2
E
F
Bak
25kDa
Bax
20kDa
HL-60/
CAFdA30
HL-60/
ara-C10
HL-60/
CAFdA4
HL-60
HL-60/
CAFdA30
HL-60/
CAFdA4
HL-60
HL-60/
ara-C10
G
Actin
42kDa
HL-60/
CAFdA30
HL-60
HL-60/
CAFdA4
HL-60/
ara-C10
